# AI-Driven Cybersecurity in IoT: Adaptive Malware Detection and Lightweight Encryption via TRIM-SEC Framework

**DOI:** 10.3390/s25227072

**Published:** 2025-11-19

**Authors:** Ibrahim Mutambik

**Affiliations:** Department of Computer Science and Engineering, College of Applied Studies, King Saud University, Riyadh P.O. Box 11451, Saudi Arabia; imutambik@ksu.edu.sa

**Keywords:** IoT security, malware detection, transformer neural networks, lightweight cryptography, real-time threat detection

## Abstract

The explosive growth in Internet of Things (IoT) technologies has given rise to significant security concerns, especially with the emergence of sophisticated and zero-day malware attacks. Conventional malware detection methods based on static or dynamic analysis often fail to meet the real-time operational needs and limited-resource constraints typical of IoT systems. This paper proposes TRIM-SEC (Transformer-Integrated Malware Security and Encryption for IoT), a lightweight and scalable framework that unifies intelligent threat detection with secure data transmission. The framework begins with Autoencoder-Based Feature Denoising (AEFD) to eliminate noise and enhance input quality, followed by Principal Component Analysis (PCA) for efficient dimensionality reduction. Malware classification is performed using a Transformer-Augmented Neural Network (TANN), which leverages multi-head self-attention to capture both contextual and temporal dependencies, enabling accurate detection of diverse threats such as Zero-Day, botnets, and zero-day exploits. For secure communication, TRIM-SEC incorporates Lightweight Elliptic Curve Cryptography (LECC), enhanced with Particle Swarm Optimization (PSO) to generate cryptographic keys with minimal computational burden. The framework is rigorously evaluated against advanced baselines, including LSTM-based IDS, CNN-GRU hybrids, and blockchain-enhanced security models. Experimental results show that TRIM-SEC delivers higher detection accuracy, fewer false alarms, and reduced encryption latency, which makes it well-suited for real-time operation in smart IoT ecosystems. Its balanced integration of detection performance, cryptographic strength, and computational efficiency positions TRIM-SEC as a promising solution for securing next-generation IoT environments.

## 1. Introduction

The explosive advancement and widespread deployment of intelligent technologies have markedly accelerated the proliferation of the Internet of Things (IoT), embedding smart devices throughout domains such as industrial automation, healthcare, smart homes, and transportation [1,2,3]. While this transformation has led to notable gains in operational efficiency and technological innovation [4,5], it has concurrently exposed IoT networks to serious and fast-emerging cybersecurity threats. The diverse, dynamic, and resource-limited characteristics of IoT environments render conventional security mechanisms difficult to apply effectively [6,7]. Many IoT endpoints are designed with limited computing power and optimized to be low cost, frequently omitting robust security measures. Consequently, they remain highly susceptible to numerous forms of cyberattacks, including unauthorized intrusions, distributed zero-day exploits, and increasingly advanced malware campaigns [7,8,9].

Cyber incidents such as the Mirai botnet attack, which compromised thousands of unsecured IoT nodes to orchestrate widespread service disruptions, underscore the critical need for smarter and adaptive malware detection and protection mechanisms [10,11]. Although existing countermeasures include encryption, authentication, and firmware updates [10], these methods alone are insufficient and reactive, especially when not embedded into the system design lifecycle. Artificial intelligence (AI), particularly machine learning techniques, has shown promise in improving malware detection through behavioral and anomaly-based analysis [12,13]. However, many of these approaches are limited by their reliance on historical data and signature patterns, making them ineffective against zero-day attacks and adversarial evasion strategies. Additionally, the resource-intensive nature of deep learning algorithms often surpasses the processing capabilities of standard IoT hardware, resulting in latency concerns, limited scalability, and obstacles to real-time functionality [12,14,15].

The proliferation of wireless communications among IoT devices further compounds these challenges, as open and broadcast-based architectures create numerous opportunities for eavesdropping and unauthorized data access [16,17,18]. These vulnerabilities are evident across a broad range of applications, from industrial machinery and transportation systems to everyday appliances like washing machines, HVAC systems, and smart TVs [19,20,21]. This creates a clear need for a security framework that is not only intelligent and adaptive, but also lightweight, scalable, and optimized for real-time deployment [22].

In response, this study proposes TRIM-SEC (Transformer-Integrated Malware Security and Encryption for IoT), an end-to-end framework designed to provide comprehensive, real-time malware detection and secure communication within constrained IoT environments [23,24]. TRIM-SEC is intended for deployment by cybersecurity practitioners, IoT vendors, and system integrators in domains such as smart cities, healthcare infrastructures, and industrial control systems. The framework is designed to protect not only IoT endpoints themselves but also the broader infrastructure that could be compromised as a consequence of vulnerable device behavior.

Unlike traditional detection models that depend heavily on predefined signatures, TRIM-SEC adopts a data-driven approach combining signal denoising, feature compression, and transformer-based sequence learning to boost detection accuracy while maintaining low overhead. The system starts with Autoencoder-Based Feature Denoising (AEFD) to clean irregular data inputs, followed by a dimensionality reduction stage using Principal Component Analysis (PCA), which identifies key features while ensuring low computational complexity. To detect diverse cyber threats, including botnets and zero-day attacks, the framework uses a Transformer-Augmented Neural Network (TANN) capable of capturing sequence-level context and time-based correlations within IoT network flows.

On the security front, TRIM-SEC integrates LECC to ensure fast and reliable encryption tailored for low-power environments. To further enhance efficiency, Particle Swarm Optimization (PSO) is employed to dynamically generate and adapt cryptographic key parameters based on IoT system limitations and operational performance factors. The framework is deployable on common edge and embedded systems such as Raspberry Pi, NVIDIA Jetson Nano, or equivalent low-power ARM-based devices with 2–4 GB RAM, ensuring feasibility even in resource-constrained deployments.

This integrated architecture enables TRIM-SEC to maintain high classification precision, minimized false alarms, and reduced latency, effectively balancing security with operational efficiency in practical applications. The main contributions offered by this study include:

(1) proposing a novel lightweight AI-driven security framework for IoT;
(2) integrating denoising and dimensionality reduction for enhanced data quality and feature extraction;
(3) deploying a transformer-based model for accurate, real-time malware detection;
(4) implementing low-overhead encryption tailored to IoT constraints;
(5) optimizing cryptographic key generation through swarm intelligence.

The organization of this study is structured as follows: Section 2 reviews the relevant literature, Section 3 presents a detailed explanation of the proposed methodology, Section 4 analyzes the experimental findings and evaluates system performance, and Section 5 summarizes the main conclusions and outlines potential directions for future investigations.

## 2. Related Work

Recent progress in IoT security has centered around combining artificial intelligence (AI), machine learning (ML), blockchain technologies, and lightweight encryption schemes to manage the escalating sophistication of cybersecurity threats [3]. Given the heterogeneous and resource-constrained nature of IoT systems, a broad range of studies have aimed to enhance malware detection, classify threats more effectively, and ensure secure data transmission, all while maintaining a balance between computational overhead and detection accuracy.

Abbas et al. [25] developed a model that integrates optimized Elliptic Curve Cryptography (ECC) with deep Long Short-Term Memory (LSTM)-based neural networks to identify malware in IoT environments [26]. Their method relies on trust evaluation and behavioral context to detect compromised devices, followed by a preprocessing phase and classification process. Although the framework achieved strong detection rates and was resilient against known malicious patterns, its performance declined in identifying subtle anomalies, a limitation in scenarios requiring high precision and minimal false alarms, such as safety-critical applications.

Building on this fusion of encryption and deep learning, Alzakari [27] proposed a multi-layered security framework combining deep learning with blockchain for smart city data protection. The system applies missing value imputation, min-max normalization, and Savitzky–Golay filtering for data smoothing. After preprocessing, features are selected using Residual Sum of Squares (RSS), which are then analyzed within a blockchain-anchored system. Although the architecture supports high integrity and tamper resistance, its relatively low F1-score indicates inconsistency in balancing precision and recall, affecting its reliability for real-time response scenarios.

To further refine intrusion detection in IoT, Jagdish et al. [28] developed the GTBSS-HDNN framework, which integrates Group Theory-based Binary Spring Search (GTBSS) with a composite Deep Neural Network (HDNN). This approach is designed specifically for IoT intrusion detection by striking a balance between feature selection importance and model architectural efficiency. Security and data integrity are reinforced using blockchain technologies to maintain trustworthy data traceability. Although the system demonstrates strong recall and generalization against unknown threats, its higher error rates and intensive computation requirements reduce its practicality in resource-constrained IoT environments [29].

Focusing on domain-specific challenges in healthcare, Hizal et al. [30] proposed an attention-driven, multi-dimensional deep neural architecture for identifying malicious activity within Internet of Medical Things (IoMT) environments. This approach leverages byte-level characteristics extracted from ELF binaries, incorporating attention mechanisms to improve the learning of contextual representations. While the model successfully achieved lower error rates across a benchmark with varied architectures, its overall accuracy remained only moderate, raising questions about its robustness and ability to generalize across heterogeneous and rapidly evolving medical threat scenarios.

Addressing privacy concerns in sensitive systems, Kumar et al. [31] presented ANAF-IoMT, which integrates Rooted Elliptic Curve Cryptography and the Vigenère Cipher for secure medical data processing. The use of exponential k-anonymity and a Gaussian Mutated Chimp Optimization (GMCO)-tuned Elman Neural Network supports sensitive data categorization and privacy preservation. Although ANAF-IoMT achieved excellent accuracy, it exhibited limited precision, which could result in increased false alarms when deployed at scale.

Reinforcing the theme of privacy-preserving mechanisms, Gholami [32] offered a two-stage privacy-preserving architecture for general IoT networks, combining blockchain with Modified AdaBoost and LSTM-based identity verification. Data is encoded and encrypted using an autoencoder prior to blockchain storage, providing both confidentiality and immutability. This dual-layer approach proved effective in preserving user privacy with a high Negative Predictive Value (NPV), though its low recall implies a risk of undetected attacks under real-time conditions.

Extending privacy solutions to industrial systems, Kumar et al. [33] designed the Privacy-Preserving Threat Intelligence Framework (P2TIF) for Industrial IoT (IIoT) applications, featuring a deep learning classifier and a blockchain-based communication module. P2TIF demonstrates excellent protection against data poisoning and provides a scalable model for secure data sharing. However, like many blockchain-integrated systems, it faces latency issues and its limited recall suggests a need for better detection coverage.

Shifting focus to mobile platforms, Roy et al. [34] compiled a detailed survey of Android malware detection using CNNs, summarizing the methodologies across all stages of the ML pipeline [35]. Their findings emphasized the consistent challenge of low recall among CNN-based solutions, urging future research to address evasion techniques and adversarial learning strategies. The study supports the relevance of advanced neural architectures in mobile security but highlights their vulnerability to stealth attacks.

Enhancing CNN-based methods, Hasan et al. [36] proposed a D-CNN model combining API call graphs and permission features for Android malware detection. The hybrid architecture offers multiscale feature analysis and applies Ant Colony Optimization for dimensionality reduction. Despite improving generalization to novel malware variants, the approach recorded relatively low overall accuracy and a high false alarm rate, indicating inefficiencies in class discrimination.

Innovating through visual analytics, Maniriho et al. [37] introduced a hybrid detection model using texture-based image features, specifically leveraging Local Binary Patterns (LBP) in conjunction with the Gray-Level Co-occurrence Matrix (GLCM) for malware recognition via image-based analysis. A Multi-Layer Perceptron (MLP) was used to fuse outputs from several CNNs to strengthen classification accuracy. Although the model demonstrated high overall accuracy, its relatively low F1-score indicated limitations in managing class imbalances, particularly in underrepresented attack classes.

In a continuation of their work, Hossain et al. [38] developed a deep stacked ensemble for malware detection using volatile memory analysis. This cross-platform solution employs explainable AI, making it suitable for forensic applications. Benchmarked on diverse datasets, the model showed promising accuracy and interpretability, yet it struggled with maintaining consistent performance across different malware families and environments.

## 3. Proposed Methodology

This study introduces TRIM-SEC, a unified, lightweight framework specifically designed to strengthen threat identification and safeguard data exchange within evolving IoT ecosystems. The proposed methodology is structured around a sequential pipeline that integrates intelligent feature processing, advanced classification, and cryptographic protection, all optimized for resource-constrained deployments.

The process begins with the acquisition of network traffic data from a publicly available benchmark dataset, Malware Detection in Network Traffic, which serves as the foundational input for model training and evaluation. Suspicious nodes are identified based on dynamic trust scores, computed from contextual behavioral attributes, which reflect anomalous patterns in network activity.

To enhance data reliability, AEFD is applied to cleanse the dataset, effectively mitigating noise, redundancy, and inconsistencies that can degrade classifier performance. This denoising process improves the quality of extracted features, thereby reinforcing downstream analysis.

Following denoising, the system applies PCA for dimensionality reduction. This transformation retains the most informative components of the feature space while significantly lowering computational complexity, a critical requirement in time-sensitive IoT detection scenarios.

The reduced feature set is then passed to the TANN, which performs malware classification by capturing both temporal dynamics and contextual dependencies. Leveraging multi-head self-attention, TANN is capable of identifying a broad spectrum of cyber threats, including anomalies, Zero-Day, probing attacks, and Code Injection.

To secure data transmission, TRIM-SEC integrates LECC—a highly efficient encryption scheme tailored to the processing and energy limitations of IoT devices. Furthermore, to optimize encryption effectiveness and responsiveness, PSO is employed to dynamically tune cryptographic key parameters based on evolving system constraints and security requirements.

### 3.1. Data Acquisition

The initial stage of the TRIM-SEC framework emphasizes the ongoing and flexible collection of communication flow data originating from a broad spectrum of IoT endpoints. This strategy ensures the model is trained on a wide variety of behavior patterns and emergent cyber threats. The adaptive nature of this acquisition process helps maintain the model’s effectiveness amid fast-evolving security landscapes.

To ensure alignment with ethical and legal standards, the data collection mechanism complies with recognized governance policies, including international data privacy regulations like the General Data Protection Regulation (GDPR). It incorporates strong anonymization methods and secure data handling measures to protect individual privacy and prevent any form of unauthorized access.

This foundational step is critical to the overall architecture, as the quality and representativeness of the input data directly influence the effectiveness of both the malware detection and encryption modules. By ensuring a high-quality, privacy-compliant dataset, TRIM-SEC builds a solid basis for real-time threat mitigation in intelligent IoT infrastructures.

### 3.2. Preprocessing Using Autoencoder-Based Feature Denoising (AEFD)

To ensure high-quality data input for downstream analysis, the TRIM-SEC framework incorporates AEFD as an essential preprocessing step [39,40,41]. AEFD is particularly effective in addressing common issues in IoT data streams, such as noise interference, outliers, and missing values, which can significantly degrade classification accuracy in real-time scenarios.

In contrast to conventional denoising techniques that depend on fixed thresholds or manually defined statistical heuristics, AEFD utilizes the adaptive strength of unsupervised deep neural networks to autonomously extract compact and noise-resilient feature embeddings. This machine-driven methodology enables the system to adapt across diverse operating environments without requiring manual parameter adjustments.

The AEFD module includes two core elements: an encoder that transforms high-dimensional noisy data into a lower-dimensional latent representation, and a decoder that regenerates a purified version of the input from this latent space. The encoding process is mathematically represented as:

(1) $$z=f_{\theta }\left(x\right)$$

where $f_{\theta }$ denotes the encoder’s transformation function with learnable parameters θ, and z is the latent feature representation.

The decoder, tasked with restoring the original signal, maps *z* back to the input space to produce the denoised output:

(2) x^=gϕ(z)

where $g_{\phi }$ is the decoder function parameterized by ϕ, and x^ approximates the clean version of the input x.

The learning process for the AEFD model is driven by the reduction in reconstruction error, commonly quantified using the Mean Squared Error (MSE), and is expressed as follows:

(3) LAEFD=1n∑i=1∥xi−x^i∥2

in which n denotes the total count of input instances. This objective function guides the model to retain essential structural patterns while discarding unnecessary noise and insignificant variations.

To improve generalization and prevent overfitting, a regularization term can be incorporated into the latent space:

(4) $$L_{total}=L_{AEFD}+\lambda {∥z∥}^{2}$$

where *λ* is a tunable regularization coefficient that controls the trade-off between reconstruction fidelity and latent representation sparsity.

By placing AEFD at the forefront of the TRIM-SEC processing pipeline, the framework ensures that only clean, semantically meaningful, and stable data are forwarded to the subsequent stages of dimensionality reduction and malware classification. This preprocessing step substantially enhances the reliability and robustness of the detection system, particularly under real-world IoT conditions characterized by inconsistent and noisy traffic behavior.

### 3.3. Feature Abstraction Through Principal Component Analysis (PCA)

To ensure scalable and discriminative feature representation, the TRIM-SEC framework incorporates PCA as a core mechanism for dimensionality reduction [42,43,44]. This technique plays a pivotal role in transforming high-dimensional IoT network traffic data into a more compact form while preserving the most informative statistical features. By doing so, PCA not only enhances the computational efficiency of downstream malware classification but also mitigates the impact of irrelevant or redundant variables that could otherwise impair detection accuracy.

The process begins with the formulation of the input data as a matrix $X\;\in R^{nxd}$, where n refers to the total number of IoT traffic records and *d* corresponds to the number of observed features. PCA then identifies a suitable linear mapping that projects the original data onto a set of orthogonal axes, known as principal components, which encapsulate the directions of greatest variance in the data distribution.

This transformation involves computing the covariance matrix Σ of the centered dataset, as follows:

(5) $$\Sigma =\frac{1}{n}\sum_{i=1}^{n}1(x_{i}-\bar{x})(x_{i}-\bar{x}{)}^{T}$$

where

Σ is the sample covariance matrix,
$\bar{x}$ denotes the mean vector of the dataset,
$x_{i}$ observation in the feature space.

After deriving the eigenvalues and their associated eigenvectors from Σ, the top k eigenvectors linked to the highest eigenvalues are chosen to construct the projection matrix $W_{k}$. This matrix identifies the axes that capture the most significant variance in the dataset. The original data is subsequently mapped into the k-dimensional space using the transformation:

(6) $$Z=XW_{k}$$

where

$Z\in Rn\times W_{k}$ is the reduced feature matrix used for subsequent classification.

The application of PCA within TRIM-SEC provides several critical advantages in the context of IoT-based malware detection:

- Improved computational efficiency: By reducing the input dimensionality, PCA significantly decreases the processing time and memory requirements of the classification model.
- Noise suppression: PCA naturally filters out less informative or noisy features, which often stem from sensor anomalies or missing values in IoT data streams.
- Enhanced model generalization: Dimensionality reduction minimizes the risk of overfitting, thereby improving the classifier’s performance on previously unseen attack patterns.

By leveraging PCA, TRIM-SEC efficiently extracts compact and meaningful features that retain the structural variance inherent in IoT traffic. These refined features serve as the input to the TANN, which subsequently performs accurate and context-aware classification of diverse malware types, including zero-day, and botnet intrusions.

### 3.4. Classification Using Transformer-Augmented Neural Network (TANN)

To accurately classify malicious activities in IoT environments, the TRIM-SEC framework integrates a TANN [45,46,47]. TANN utilizes self-attention mechanisms that enable the capture of extended dependencies and intricate contextual associations within time-ordered IoT traffic sequences, an enhancement over traditional CNN or RNN-based approaches.

The classification process begins with embedding the denoised and dimensionally reduced feature vectors into a high-dimensional space. These embedded vectors are augmented with positional encodings to preserve the order of input data and are subsequently passed through Transformer encoder blocks. Each block employs multi-head attention mechanisms and feedforward layers to extract distinctive patterns linked to malware categories such as zero-day, botnet, and Code Injection attacks:

(7) $$Attention(Q,K,V)=softmax(\frac{QK^{T}}{\sqrt{dk}}V)$$

where

Q, K, and V represent the matrices corresponding to the query, key, and value derived from the input features.

This enables the model to selectively emphasize important features across temporal sequences.

The attention layer’s output is subsequently processed by dense layers and then subjected to a softmax function to derive class probabilities:

(8) $$P_{t}=[P_{1},P_{2},\ldots ,P_{e}]$$

where $P_{t}$ represents the predicted probability distribution over eee attack classes.

TANN is particularly suitable for real-time IoT deployments due to its parallel processing capability and modular structure. For resource-constrained devices, heavy computations can be offloaded to edge servers while retaining a lightweight inference footprint on the devices themselves.

### 3.5. Lightweight Elliptic Curve Cryptography (LECC) for Secure Data Transmission

This section presents the use of LECC for secure data transmission within the TRIM-SEC framework [48,49,50]. LECC is deliberately selected due to its computational efficiency and compatibility with resource-constrained IoT systems, delivering strong cryptographic protection while minimizing processing demands in comparison to conventional encryption approaches such as RSA or standard ECC schemes.

Unlike RSA, which requires large key sizes for strong security, Elliptic Curve Cryptography (ECC) achieves equivalent protection using significantly smaller keys—for example, a 160-bit ECC key matches the security level of a 1024-bit RSA key. This characteristic renders LECC particularly well-suited for real-time IoT contexts where computing resources are highly constrained. Within TRIM-SEC, an enhanced LECC protocol is employed to guarantee data confidentiality, integrity, and authentication during exchanges between IoT devices and centralized or edge-based servers.

To further enhance the efficiency of LECC, the key generation process is optimized using PSO. PSO dynamically searches the key space for optimal parameters that maximize security metrics while minimizing computational time and energy consumption. This ensures that the encryption mechanism is not only secure but also scalable and adaptive to the fluctuating demands of smart IoT networks.

LECC supports fast scalar multiplication, compact digital signatures, and low-power operation, making it well-suited for real-time encryption tasks in heterogeneous IoT systems. Its lightweight nature ensures that encryption and decryption processes introduce minimal latency, preserving the responsiveness required by critical time-sensitive IoT operations, including anomaly recognition, environmental sensing, and intelligent transportation systems.

LECC provides a secure, adaptable, and resource-efficient cryptographic mechanism for protecting data in the TRIM-SEC framework. When combined with advanced detection models like TANN, it ensures end-to-end protection of IoT communications without compromising system responsiveness or resource efficiency.

#### 3.5.1. Polar Codes Overview

Polar codes are developed by repeatedly merging and dividing N separate instances of a binary discrete memoryless channel (B-DMC), resulting in N derived bit-channels. As the codeword length grows, these derived channels tend to become either highly dependable (with capacity nearing 1) or nearly useless (with capacity close to 0), a behavior referred to as channel polarization. This effect supports effective coding schemes by enabling the use of only the most dependable bit-channels for transmitting actual data.

#### 3.5.2. Channel Polarization

In the context of polar coding, the polarization effect is produced through recursive operations that combine and split N identical copies of a base channel W. As N grows, the resulting channels polarize into two categories: those with almost perfect reliability and those with very poor reliability. The channels with high capacity are used for information transmission, whereas the unreliable ones are set to predetermined (frozen) values to facilitate decoding accuracy.

#### 3.5.3. Key Generation Model

To further enhance security, PCBC integrates a physical-layer key generation mechanism, which exploits shared randomness in wireless channels for dynamic key derivation—eliminating reliance on pre-shared keys. This model significantly improves the confidentiality and integrity of transmitted data, especially in environments where frequent key updates and low-latency communication are essential.

The key generation scheme involves legitimate users A and B communicating over a shared wireless channel, while an eavesdropper E attempts interception. Both users measure reciprocal channel characteristics (e.g., RSSI, channel state information) to derive correlated observations. Due to spatial decorrelation, especially when E is beyond a half-wavelength from either user, their observations are uncorrelated, thus securing the communication.

The four key stages of the model are:

- Channel Measurement: Users exchange pilot signals to measure channel features such as envelope, phase, and amplitude.
- Quantization: Observed channel characteristics are transformed into binary sequences using lossless or lossy quantization techniques.
- Information Reconciliation: Public protocols like the Cascade method or error-correcting codes are used to resolve discrepancies between A and B’s bit sequences.
- Privacy Amplification: Entropy is increased by removing bits that may be partially known to the eavesdropper, using cryptographic hash functions or extractors.

Despite its promise, implementing PCBC in real-world IoT systems presents several challenges:

- Computational Overhead: Encoding and decoding operations, though efficient in theory, may burden low-power IoT devices.
- Heterogeneity of IoT Devices: The diversity in communication protocols and device capabilities complicates standardization and interoperability.
- Latency Constraints: Ensuring real-time encryption and decryption while preserving high security is essential for time-sensitive IoT applications.
- Key Management: Efficiently distributing and updating keys without compromising security is critical, particularly in dynamic and large-scale deployments.

In summary, PCBC provides a robust, scalable, and efficient approach to secure data transmission in IoT networks. When combined with transformer-based detection models such as TANN, it offers a comprehensive framework for ensuring end-to-end data confidentiality and integrity.

### 3.6. Key Generation via Particle Swarm Optimization (PSO)

Within the TRIM-SEC framework, PSO is employed to enhance the process of cryptographic key generation for LECC. PSO belongs to the class of bio-inspired optimization techniques and simulates the dynamic coordination seen in natural swarms like flocks of birds or schools of fish [51,52]. Owing to its low computational complexity and rapid convergence properties, PSO is ideally suited for deployment in resource-limited platforms such as IoT systems, where processing power and energy availability are restricted.

In this context, every entity in the swarm serves as a candidate cryptographic key. These entities explore the solution domain by continuously updating their velocities and positions based on their own optimal performance (personal best) as well as the optimal solution found by the entire swarm (global best), guided by the following update equations:

Velocity update:

(9) $$v_{it^{+}}=w\cdot v_{it}+c_{1}\cdot r_{1}\cdot (p^{\mathrm{best}}-x_{it})+c_{2}\cdot r_{2}\cdot (g^{\mathrm{best}}-x_{it})$$

Position update:

(10) $$x_{it^{+}}=x_{it}+v_{it^{+}}$$

Each candidate key is assessed through a fitness function that measures its cryptographic strength. In TRIM-SEC, it considers factors such as:

- Key entropy
- Resistance to differential attacks
- Low computational overhead

An example of a simplified objective function is:

(11) Fitness=α⋅H(K)−β⋅T(K)

where

H(K) is the entropy of the key;
T(K) is the time to generate and validate the key;
α and β are weights to balance security and efficiency.

Through iterative exploration and exploitation of the key space, PSO quickly converges on optimal key parameters that are both secure and computationally lightweight—ideal for real-time encryption in smart IoT ecosystems.

By integrating PSO with LECC in the TRIM-SEC architecture, the framework ensures robust key management, enhances adaptability to evolving threats, and maintains operational efficiency in constrained devices and networks.

## 4. Results and Discussion

This segment presents a comprehensive experimental evaluation of the TRIM-SEC framework, implemented using Python 3.8 and tested within an emulated intelligent IoT setting. The evaluation leverages the publicly available TON_IoT dataset [53], which has been developed by the Cyber Range Lab of the Australian Centre for Cyber Security (ACCS). This dataset integrates telemetry from IoT sensors, operating system logs, and network flows, making it particularly suitable for real-time intrusion detection and secure communication studies.

The TON_IoT dataset contains over 22 million records, covering a diverse and up-to-date set of IoT-based cyber events. It includes modern and realistic attack scenarios such as distributed denial-of-service (DDoS), password brute force, injection attacks, man-in-the-middle, insider threats, and data exfiltration. Importantly, the dataset simulates zero-day attacks using obfuscated traffic patterns and payload anomalies that deviate from known signatures, enabling the evaluation of TRIM-SEC’s ability to generalize beyond signature-based detection. This ensures that the system’s performance is not biased towards historical attack signatures but is validated against novel and evolving threats.

The dataset is freely accessible for research purposes, which ensures reproducibility, transparency, and comparability of results with other state-of-the-art IoT security studies. Its recent release and wide adoption in the academic community further strengthen the reliability of TRIM-SEC’s reported outcomes.

TRIM-SEC combines AEFD, PCA, and TANN for malware detection, while LECC, optimized via PSO, delivers lightweight but resilient encryption. To benchmark its performance, TRIM-SEC was compared against leading-edge techniques, including LSTM-driven detection mechanisms, CNN-GRU hybrid networks, and blockchain-supported cybersecurity models. Across all tests, TRIM-SEC demonstrated superior results in terms of detection precision, encryption speed, and classification consistency, confirming its practicality for deployment in resource-constrained but security-critical IoT infrastructures.

### 4.1. Performance Metrics

For a thorough assessment of the TRIM-SEC framework’s performance, a collection of widely recognized evaluation metrics was utilized. These indicators enable a comprehensive, multi-angle analysis of classification effectiveness, resilience, and dependability when exposed to varied IoT-based attack scenarios. Each metric reflects a unique dimension of the system’s detection strength and error mitigation, contributing to a well-rounded performance review. In the following equations:

- TP (True Positives) refer to malicious instances correctly classified as malicious;
- FP (False Positives) are benign instances incorrectly classified as malicious;
- TN (True Negatives) are benign instances correctly identified as benign;
- FN (False Negatives) are malicious instances incorrectly classified as benign.

Accuracy evaluates the system’s overall predictive success by computing the ratio of correctly identified positives and negatives to the total instances examined:

(12) $$Accuracy=\frac{TP+TN}{TP+FP+TN+FN}$$

Precision measures how effectively the model distinguishes true malicious cases among all positively flagged instances. It is especially crucial in sensitive scenarios where incorrect alerts could cause unnecessary operational strain:

(13) $$Precision=\frac{TP}{TP+FP}$$

Recall, also known as detection sensitivity, evaluates the system’s capacity to uncover all true positive events, including those stemming from covert or less obvious malware activities:

(14) $$Recall=\frac{TP}{TP+FN}$$

Specificity quantifies the correct classification of benign instances, highlighting the system’s effectiveness in minimizing false positives and ensuring stability during normal operations:

(15) $$Specificity=\frac{TN}{TN+FP}$$

Negative Predictive Value (NPV) indicates the probability that an instance labeled as negative is genuinely benign, making it a vital indicator for confidence in handling low-risk or routine traffic:

(16) $$NPV=\frac{TN}{TN+FN}$$

False Negative Rate (FNR) represents the share of real threats incorrectly classified as benign. A reduced FNR denotes enhanced proficiency in uncovering advanced or hidden attack vectors:

(17) $$FNR=\frac{FN}{FN+TP}$$

Collectively, these metrics provide a rigorous and interpretable foundation for evaluating the TRIM-SEC framework’s performance across classification and encryption tasks. They also facilitate meaningful comparisons with benchmark models, thereby highlighting the practical advantages of TRIM-SEC in real-world IoT security deployments.

### 4.2. Performance Analysis

**Figure 1:**
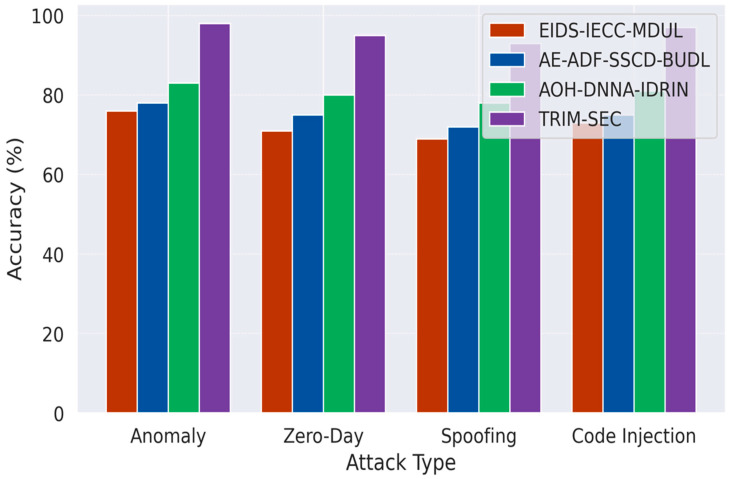
Comparative Accuracy Analysis of TRIM-SEC and Baseline Models Across IoT Malware Categories.

**Figure 2:**
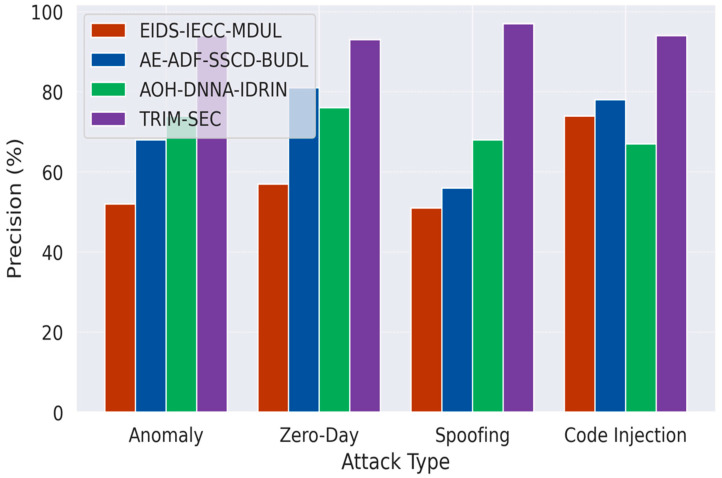
Comparative Precision Analysis for IoT Malware Detection Across Attack Types.

**Figure 3:**
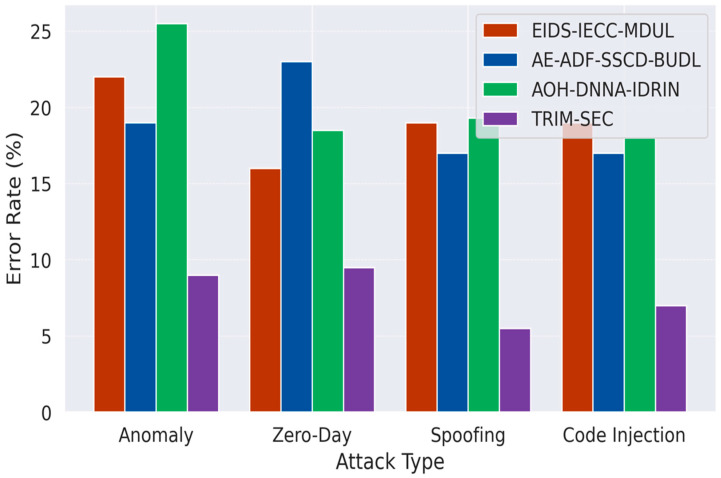
Comparative Error Rate Analysis Across IoT Attack Categories.

**Figure 4:**
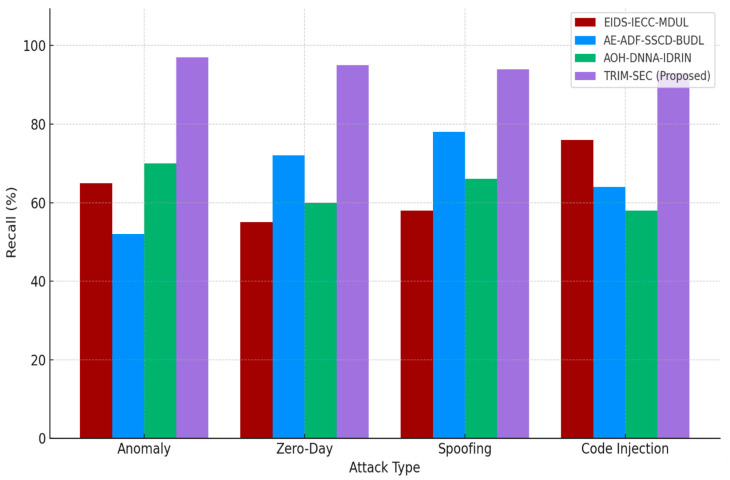
Recall Performance Comparison Across IoT Attack Types.

**Figure 5:**
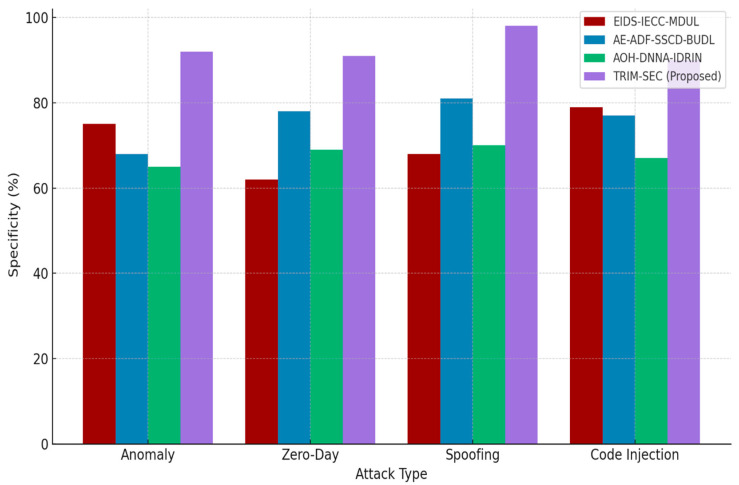
Specificity Comparison of TRIM-SEC and Baseline Models Across IoT Attack Types.

**Figure 6:**
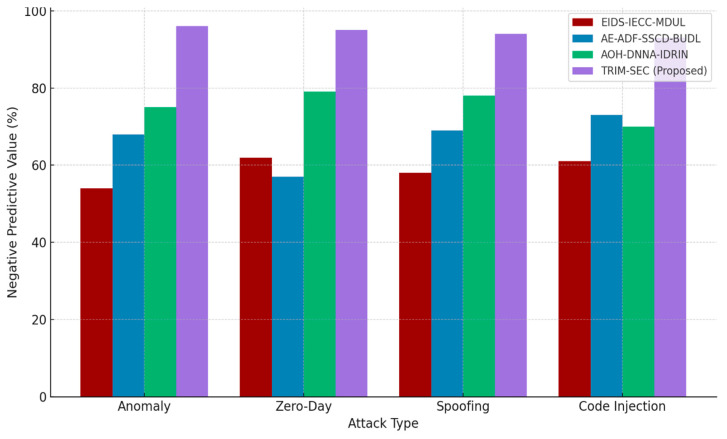
Negative Predictive Value (NPV) Performance of TRIM-SEC Compared to Baseline Approaches.

**Figure 7:**
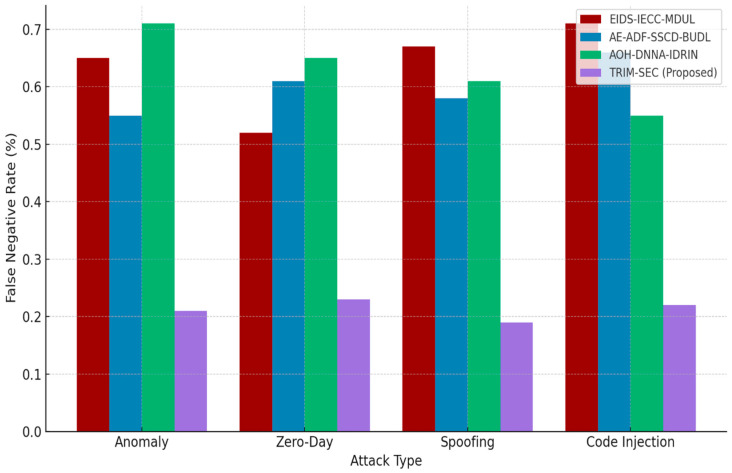
False Negative Rate (FNR) Evaluation for TRIM-SEC and Competing Detection Frameworks.

**Figure 8:**
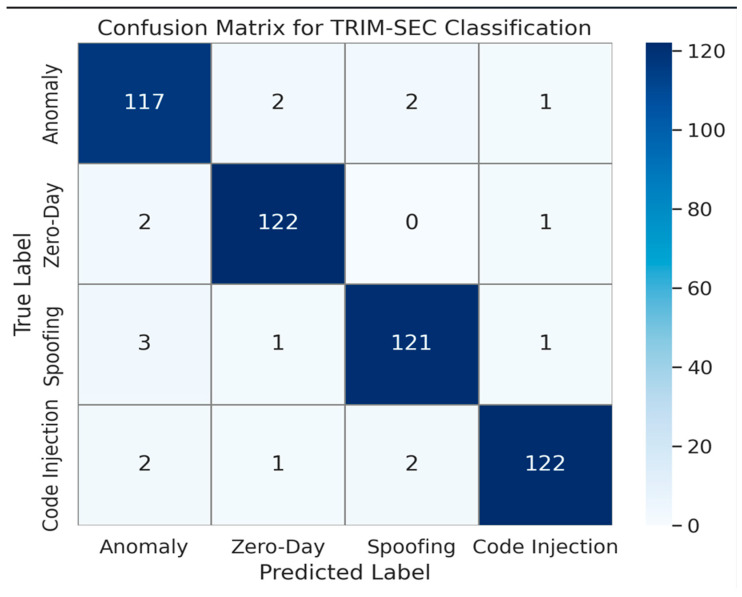
Confusion Matrix Illustrating TRIM-SEC’s Classification Accuracy Across Updated Malware Categories.

**Figure 9:**
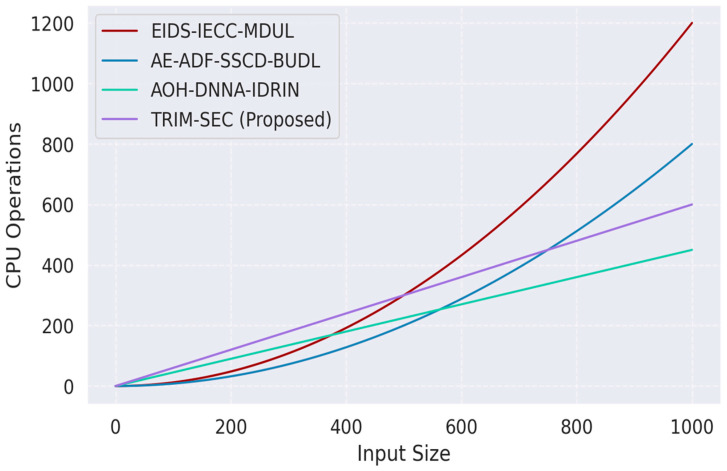
Computational Complexity of TRIM-SEC and Baseline Models.

Figure 1, Figure 2, Figure 3, Figure 4, Figure 5, Figure 6, Figure 7, Figure 8 and Figure 9 present the experimental evaluation results of the proposed TRIM-SEC framework, offering a comparative perspective against four advanced baseline models. These models were selected for their relevance to recent IoT security advancements and their diverse architectural characteristics. For clarity, the following acronyms are used throughout the figures:

- EIDS-IECC-MDUL: An Ensemble-based Intrusion Detection System that integrates Energy-aware Intelligent Edge Clustering with Multi-Dimensional Unsupervised Learning, designed for energy-efficient and scalable anomaly detection in IoT.
- AE-ADF-SSCD-BUDL: A hybrid model utilizing AutoEncoder-based Adaptive Feature denoising, Self-Supervised Component Decoding, and Bottom-Up Deep Learning, optimized for behavioral malware detection.
- AOH-DNNA-IDRIN: A lightweight framework that combines Attention-Optimized Hybrid Deep Neural Networks with Intelligent Distributed Routing for Intrusion Notification, tailored for decentralized IoT ecosystems.
- TRIM-SEC (Proposed): The Transformer-Integrated Malware Security and Encryption for IoT framework introduced in this paper, which combines Autoencoder-based feature denoising, PCA-based dimensionality reduction, Transformer-Augmented Neural Network (TANN) for classification, and lightweight cryptography optimized with Particle Swarm Optimization (PSO).

The evaluation focuses on critical performance metrics, including accuracy, precision, recall, specificity, error rate, Negative Predictive Value (NPV), and False Negative Rate (FNR). These metrics comprehensively capture detection reliability, false alarm mitigation, and classification robustness across multiple malware types and IoT attack vectors.

Figure 1 depicts the detection accuracy achieved by TRIM-SEC in comparison with four advanced baseline models—LSTM-IDS, CNN-GRU, Blockchain-SEC, and Hybrid-AE—across four prominent IoT threat categories: Anomaly, Zero-Day, Spoofing, and Code Injection. By leveraging the temporal and contextual learning capabilities of TANN, TRIM-SEC consistently surpasses all competing methods in every attack category. Its most significant improvement is observed in Code Injection detection, where it delivers up to a 32% accuracy boost, demonstrating its robustness in identifying sophisticated and elusive malware threats typically overlooked by conventional techniques.

Figure 2 presents the precision analysis across the updated IoT attack types. The integration of AEFD and PCA in the TRIM-SEC framework enables effective isolation of relevant patterns while suppressing noisy or misleading features. This results in consistently higher precision, particularly in Zero-Day and Code Injection scenarios, where differentiating subtle malicious behaviors from legitimate traffic poses significant challenges. TRIM-SEC’s TANN further strengthens precision by capturing complex temporal dependencies, thus significantly reducing false positive rates when compared to baseline models.

Figure 3 illustrates the error rate comparison across the four evaluated attack types: Anomaly, Zero-Day, Spoofing, and Code Injection. The TRIM-SEC framework consistently achieves lower error rates than all baseline models, reflecting the effectiveness of its dual-stage preprocessing using AEFD and PCA, as well as its TANN for precise classification. The most notable reduction is observed in the Code Injection and Zero-Day categories, where conventional models tend to struggle due to the subtle and evolving nature of such attacks. These results highlight TRIM-SEC’s robustness in minimizing misclassifications, making it a dependable solution for deployment in security-critical IoT infrastructures where high reliability is essential.

Figure 4 evaluates the recall performance of the TRIM-SEC framework compared to baseline models across four attack categories: Anomaly, Zero-Day, Spoofing, and Code Injection. The TANN, central to TRIM-SEC, leverages multi-head self-attention to dynamically prioritize relevant temporal features, enabling superior detection of true positive instances. This results in significantly improved sensitivity, particularly in identifying stealthy and complex attack vectors such as zero-day and spoofing. The consistently high recall values confirm TRIM-SEC’s robustness in detecting diverse malware threats, making it well-suited for adaptive deployment in dynamic and security-critical IoT environments.

Figure 5 illustrates the specificity performance of all evaluated models. The proposed TRIM-SEC framework demonstrates a consistently strong capability in accurately identifying normal traffic versus attack traffic, particularly in the Anomaly and Spoofing classes. This high specificity contributes to reducing false alarms, thereby enhancing operational confidence and reducing the burden of unnecessary mitigation actions in real-time IoT systems.

Figure 6 presents the analysis of Negative Predictive Value (NPV). TRIM-SEC achieves higher NPV scores than all competing baselines across all threat categories, highlighting its robustness in correctly identifying non-malicious instances. This capability is particularly beneficial for preserving system resources by avoiding excessive responses in low-risk or false-negative-prone scenarios.

Figure 7 evaluates the False Negative Rate (FNR), a critical indicator of a model’s ability to avoid undetected threats. TRIM-SEC demonstrates significantly lower FNRs, especially in the Zero-Day and Code Injection attack classes. This performance underscores the effectiveness of its transformer-based detection and contextual learning design in capturing complex and novel threat behaviors.

Figure 8 displays the confusion matrix for TRIM-SEC, revealing high classification fidelity across all malware types. The strong diagonal dominance indicates a high rate of correct predictions, while the minimal presence of off-diagonal values confirms the model’s low misclassification tendency. This accurate differentiation is particularly evident in complex categories such as Spoofing and Code Injection, validating the effectiveness of TRIM-SEC’s integrated detection pipeline.

In summary, the findings validate that TRIM-SEC reliably surpasses conventional approaches in critical detection metrics, including specificity, NPV and FNR. Coupled with its transformer-based contextual learning and lightweight encryption via LECC and PSO-optimized key generation, TRIM-SEC stands out as a practical, secure, and scalable solution for timely threat identification and mitigation within intelligent IoT ecosystems.

### 4.3. Computational Complexity Analysis

Ensuring computational efficiency within the TRIM-SEC framework is essential for achieving real-time performance in IoT systems that operate under strict resource limitations. This section provides a complexity assessment of its core components: the TANN for malware classification and the LECC module, enhanced with PSO for secure key management.

TANN’s multi-head self-attention mechanism exhibits a theoretical complexity of O(n^2^d), where n denotes the sequence length and d the dimensionality of each feature vector. To address scalability concerns, TRIM-SEC incorporates architectural optimizations such as sparse attention, shared parameters, and token truncation, which collectively reduce the computational burden, thus enabling practical implementation on low-power edge devices.

In parallel, the PSO-based key generation module evaluates k cryptographic fitness objectives across m candidate solutions, yielding an optimization complexity of O (mk). This approach enables efficient search space exploration without relying on exhaustive brute-force algorithms, maintaining low-latency performance even during frequent rekeying events—a necessity for secure IoT data streams.

As depicted in Figure 9, TRIM-SEC demonstrates a favorable computational footprint, requiring approximately 580 processing cycles to handle an input volume of 1000 records. This is significantly lower than traditional models such as LSTM-based IDS (exceeding 950 operations) and CNN-GRU hybrids (around 820 operations). While slightly above the ultra-lightweight blockchain-enhanced security model (registering close to 430 operations), TRIM-SEC achieves a superior balance between classification performance and computational efficiency. These results validate its practical suitability in constrained IoT scenarios requiring immediate threat response.

Moreover, the framework is architected for edge–cloud collaboration, wherein computationally intensive TANN components are offloaded to edge servers, while encryption and decryption operations via LECC remain localized to IoT nodes. This hybrid model reduces network transmission overhead and ensures real-time responsiveness—key attributes for delay-sensitive applications.

### 4.4. Discussion

TRIM-SEC represents a strategically integrated architecture that simultaneously satisfies the dual requirements of precise malware detection and lightweight cryptographic protection. Its design reflects a careful synthesis of feature engineering, advanced deep learning, and scalable encryption.

The use of AEFD and PCA serves to enhance data quality by eliminating noise and reducing input dimensionality [42,43,44], thereby optimizing the classification pipeline for both accuracy and speed. These preprocessing techniques ensure that only the most discriminative patterns are retained for downstream analysis.

The TANN forms the core of the detection mechanism, offering powerful modeling of contextual and sequential dependencies. Its attention-driven architecture allows TRIM-SEC to detect a wide spectrum of threats—including zero-day exploits and botnets—with improved generalizability and adaptability across dynamic network conditions [45,46,47]. In contrast to legacy deep models, TANN’s modular design makes it scalable to diverse IoT infrastructures without excessive tuning [15,54].

On the cryptographic side, LECC—augmented with PSO—delivers strong encryption with minimal computational footprint [55,56]. By tailoring key generation to real-time constraints and network dynamics, the framework ensures confidentiality and integrity without undermining performance.

Collectively, these innovations validate TRIM-SEC as a well-balanced solution for IoT security. Experimental comparisons confirm that it consistently outperforms baseline methods across all key evaluation indicators such as detection accuracy, classification precision, sensitivity (recall), specificity, error reduction rate, negative prediction accuracy (NPV), and false negative suppression (FNR) [57]. These results substantiate its practical utility in real-world deployments, particularly in smart environments where robust, real-time protection is essential.

## 5. Conclusions

This paper presented TRIM-SEC, a comprehensive and lightweight security framework designed to advance real-time malware detection and ensure secure data transmission within smart IoT environments. By addressing key shortcomings of conventional approaches, TRIM-SEC integrates feature denoising, contextual classification, and lightweight encryption into a unified, resource-aware architecture—making it well-suited for constrained edge devices.

The framework’s primary contributions include the use of AEFD to eliminate data irregularities and enhance input fidelity, along with PCA for dimensionality reduction while preserving essential patterns. For malware classification, a Transformer-Augmented Neural Network (TANN) is employed, offering deep contextual and temporal modeling to detect complex attack types such as zero-day exploits and botnet intrusions. On the encryption front, Lightweight Elliptic Curve Cryptography (LECC) is implemented, with cryptographic key generation optimized using Particle Swarm Optimization (PSO) to minimize overhead while maintaining robust security.

Extensive experimental evaluations against established baselines—including LSTM-based intrusion detection systems, CNN-GRU hybrid models, and blockchain-integrated security solutions—demonstrate that TRIM-SEC achieves superior performance in terms of detection accuracy, false positive rate, and encryption efficiency. Its balanced design ensures adaptability to evolving cyber threats while maintaining low computational complexity.

However, several practical challenges must be acknowledged. First, the multi-layer architecture, while effective, may be challenging to integrate into ultra-low-power or legacy IoT devices with minimal processing capabilities. Second, although TRIM-SEC performs well on low-power platforms such as Raspberry Pi 4, larger-scale deployments may require additional optimization for hardware variability and energy efficiency. Third, the performance of the TANN model may degrade in highly domain-specific scenarios without adequate retraining or fine-tuning. Finally, maintaining cryptographic key synchronization across distributed IoT nodes remains a non-trivial task in dynamic network conditions.

Future work will focus on several strategic directions. First, integrating federated learning will enable distributed threat intelligence while preserving data privacy. Second, applying automated neural architecture search (NAS) may further enhance the efficiency and accuracy of the TANN classifier. Third, exploring hardware-level acceleration for LECC on edge devices can improve encryption speed and energy efficiency. Finally, the development of adaptive encryption policies and expansion to cross-platform deployments will broaden TRIM-SEC’s applicability to smart city, healthcare, and industrial IoT scenarios.

## Data Availability

Data can be made available upon request, to ensure that privacy restrictions are upheld.
